# Enhanced Osseointegration and Bio-Decontamination of Nanostructured Titanium Based on Non-Thermal Atmospheric Pressure Plasma

**DOI:** 10.3390/ijms21103533

**Published:** 2020-05-16

**Authors:** Yuhao Zeng, Satoshi Komasa, Hisataka Nishida, Akinori Agariguchi, Tohru Sekino, Joji Okazaki

**Affiliations:** 1 Department of Removable Prosthodontics and Occlusion, Osaka Dental University, 8-1, Kuzuhahanazono-cho, Hirakata-shi, Osaka 573-1121, Japan; komasa-s@cc.osaka-dent.ac.jp (S.K.); akinori@agariguchi.com (A.A.); joji@cc.osaka-dent.ac.jp (J.O.); 2 The Institute of Scientific and Industrial Research, Osaka University, Suita, Osaka 565-0871, Japan; hnishida@sanken.osaka-u.ac.jp (H.N.); sekino@sanken.osaka-u.ac.jp (T.S.)

**Keywords:** peri-implantitis, osseointegration, biofilm inhibition, non-thermal plasma treatment, alkali-treated titanium, nanoporous network structures

## Abstract

Alkali-treated titanate layer with nanonetwork structures (TNS) is a promising surface for improving osseointegration capacity in implants. Nevertheless, there is a risk of device failure as a result of insufficient resistance to biofilm contamination. This study tested whether treatment using a handheld non-thermal plasma device could efficiently eliminate biofilm contamination without destroying the surface nanostructure while re-establishing a surface that promoted new bone generation. TNS specimens were treated by a piezoelectric direct discharge (PDD) plasma generator. The effect of decontamination was performed utilizing *Staphylococcus aureus*. The evaluation of initial cell attachment with adhesion images, alkaline phosphatase activity, extracellular matrix mineralization, and expression of genes related to osteogenesis was performed using rat bone marrow mesenchymal stem cells, and the bone response were evaluated in vivo using a rat femur model. Nanotopography and surface roughness did not significantly differ before and after plasma treatments. Cell and bone formation activity were improved by TNS plasma treatment. Furthermore, plasma treatment effectively eliminated biofilm contamination from the surface. These results suggested that this plasma treatment may be a promising approach for the treatment of nanomaterials immediately before implantation and a therapeutic strategy for peri-implantitis.

## 1. Introduction

Titanium is one of the most prevalently applied materials for orthopedic and dental implants owing to its excellent mechanical properties, corrosion resistance, and biocompatibility [1,2]. Nevertheless, titanium implant utilization is limited by the risk of peri-implant infections, prolonged osseointegration healing time, and inadequate osteoconductive properties, particularly in patients with osteoporosis [3]. The clinical long-term success rates of titanium-based implants are 89.23% and 82.94% after 10- and 16-year follow-up periods, respectively [4]. To further increase the clinical long-term success rate of implants, the promotion of early osseointegration, long-term stability of the bone-implant interface, and reduced peri-implantitis are required [2,5,6]. The physical and chemical characteristics of the implant surface play a crucial role in early-stage bone formation around implants [7].

Owing to the susceptibility of the passive oxide layer and its electroconductivity, implant surface modifications, such as sandblasting [8], acid-etching [9], oxidation [10], and calcium phosphate deposition (alone or in combination) [11,12], have been undertaken over the past three decades to transform the roughness, micro- and nano-scale features, and chemical composition of the implant surface; these modifications can impart high levels of biocompatibility and promote early bone formation around the implants [13,14,15].

Our previous research shows that a homogeneous, hydrophilic sodium titanate layer with nanonetwork structures (TNS) is generated on the titanium surface after high-concentration alkaline treatment [16]. TNS has higher roughness and hydrophilicity compared to Ti and is, therefore, more compatible with the protein and cell attachment, as well as hydroxyapatite formation; these factors result in excellent osteogenic activity [17]. Nevertheless, several challenges must still be resolved before their clinical application. More specifically, alkali-treated titanium with nanostructures is still inadequate in terms of resistance to the bacterial attachment and biofilm formation that can eventually cause peri-implantitis [18], and more promptly, osseointegration is still a requirement in the early stage of implantation.

Plasma is one of the four fundamental states of matter and is defined as a neutral ionized gas constituted of particles in permanent interaction, which include photons, electrons, positive and negative ions, atoms, free radicals, and excited or non-excited molecules. Plasma could be obtained at atmospheric pressure by several techniques such as Radio-frequency (RF) plasmas [19], Dielectric barrier discharges (DBD) plasmas [20], Corona discharge plasmas [21], Gliding arc discharge plasmas [22,23]. As a consequence of reactive oxygen and nitrogen species (RONS) production, plasma treatment has been used to remove contamination and impart hydrophilicity to implant surfaces, which, in turn, promotes protein and cell adhesion [24,25]. According to Lee et al., plasma treatment decreases bacterial attachment by carbon-cleaning the implant surface, thereby reducing the risk of implant infection [26]. The RONS generated by plasma treatment could decontaminate and inhibit biofilm recolonization on the implant surface without destroying the elaborate surface geometry of the implant, while also promoting osteoblast attachment and differentiation [23,25,27,28]. Plasma treatment is non-toxic, low temperature, safe, and has high treatment efficiency, making it more suitable for clinical applications than other sterilization methods. The use of a handheld, nonthermal, atmospheric plasma device that utilizes piezoelectric technology has recently been focused on medical applications. As it has a higher processing efficiency and is more environmentally friendly than UV, laser, and other types of plasma treatment, the nonthermal atmospheric plasma device is very convenient for immediate treatment prior to implantation. Simultaneously, this nonthermal atmospheric plasma, which could eliminate biofilm while re-established surface characteristics that are promotive for bone regeneration, are particularly suitable for the treatment of peri-implantitis [29].

In the present study, we hypothesized that nonthermal atmospheric plasma treatment could change the chemical components on the implant surface and preserve the roughness of the nanostructured surface established by alkali treatment, thereby promoting osseointegration and decontamination. To prove this hypothesis, we investigated the effect of plasma treatment on biofilm formation, cell adhesion, and osseointegration in the early stage of implantation through in vivo and in vitro experiments. *Staphylococcus aureus* was utilized to illustrate the effect of plasma treatment on decontamination. Plasma treatment of TNS effectively changed the chemical composition on the sample surface, which further improved implant hydrophilicity and facilitated cell attachment and osteogenic differentiation, while decontaminating the biofilm without destroying the TNS surface nanomorphology. Plasma treatment has potential clinical applications, such as immediate treatment before implantation and the therapeutic treatment of peri-implantitis.

## 2. Results

### 2.1. Surface Characterization

**Figure 1:**
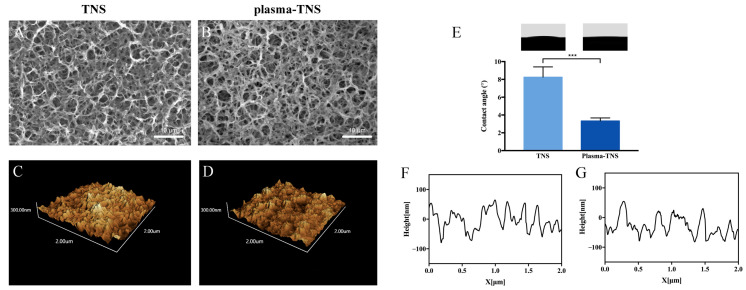
Scanning electron micrographs of (**A**) Titanate layer with nanonetwork structures (TNS) and (**B**) plasma-TNS; Scanning probe micrographs and a typical surface profile of (**C**,**F**) TNS and (**D**,**G**) plasma-TNS. (**E**) The measurement of the contact angle on the surface of TNS and plasma-TNS. Data shown are the means ±SD (*n* = 3). *** *p* < 0.001.

Scanning electron microscopy (SEM), which has made a significant contribution to the observation and characterization of nanomorphology, was utilized to survey the surface topography of TNS and plasma-TNS samples. SEM micrographs showed that the nanoporous network structure within an average diameter of 50–100 nm was well-interconnected and homogeneous on the titanium surface after alkali treatment. As shown in Figure 1, the nanoporous structure of the sample surface did not change significantly after plasma treatment. The effect of plasma treatment on the surface measured by atomic force microscopy (AFM) demonstrated similar nanotopographies on the surfaces of TNS and plasma-TNS samples (Figure 1). Furthermore, the values of surface roughness (Ra and Rz) measured by AFM indicated that there was no significant difference between TNS and plasma-TNS samples (Table 1). Hydrophilicity analysis of the surface of TNS and plasma-TNS samples showed that the TNS surface exhibited hydrophilic properties with a contact angle of approximately 9°. Notably, a significant change in wettability was recognized on the surface of plasma-TNS samples with a contact angle of <3°, which exhibited super-hydrophilic characteristics (Figure 1E).

**Figure 2:**
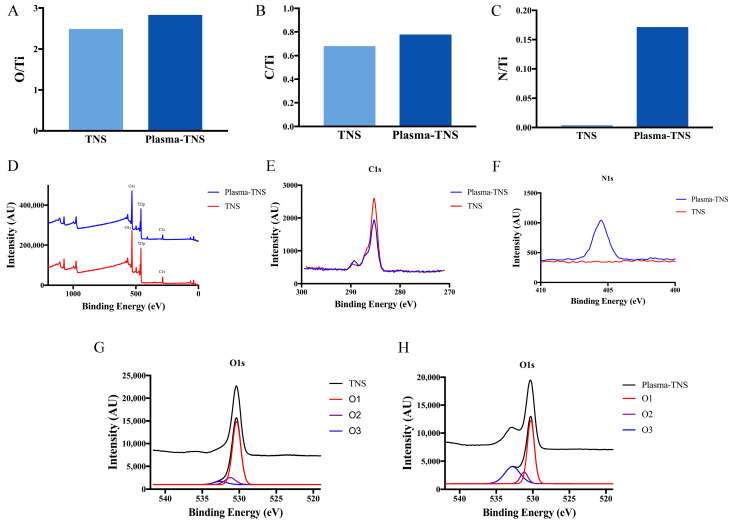
XPS analysis on the surface of TNS and plasma-TNS presented as the mean calculated from the random location of three samples: (**A**) oxygen/titanium ratio, (**B**) carbon/titanium ratio, (**C**) nitrogen/titanium ratio, (**D**) Wide-survey XPS spectra of the specimens, (**E**) high-resolution spectra of carbon 1 s, (**F**) high-resolution spectra of nitrogen 1 s, and high-resolution spectra of oxygen 1 s on the surface of (**G**) TNS and (**H**) plasma-TNS, respectively.

The chemical composition and chemical bonding on TNS and plasma-TNS surfaces were investigated by X-ray photoelectron spectroscopy (XPS). Wide-survey XPS spectra of the specimens revealed that the surface chemical composition contained the characteristic peaks of Ti, O, C, N, and Na. To further investigate the changes in chemical composition and chemical bonding after plasma treatment, high-energy-resolution spectra for C1s, O1s, N1s, and Ti2p were obtained. Ti 2p_3/2_ and Ti 2p_1/2_ components were shown at 458.5 eV and 464.2 eV, respectively, with a 5.7 eV spin-orbital splitting value consistent with the Ti^4+^ valence state. The Na1s spectra from TNS and plasma-TNS samples did not significantly differ, indicating that both sample types were covered by titanite. This was consistent with the findings of previous studies [30]. The presence of carbon contamination on all surfaces was obvious, which was also consistent with previous studies [31]. Notably, the carbon ratio on the sample surface significantly decreased after plasma treatment. Figure 2 shows the ratio of N/Ti to O/Ti on the surface of TNS and plasma-TNS samples. The ratios indicated the notable increase of O and N content on the surface of plasma-treated samples.

High-energy-resolution spectra of the N1s peak before and after plasma treatment are shown in Figure 2F. The distinct spectra observed on plasma-treated samples were exhibited at a binding energy of 406.8 eV, corresponding to NO_x_ (nitrate) species [32,33]. In contrast, few of these spectra were found on the surface of untreated samples. Figure 2 also shows the high-resolution oxygen 1s spectra from the surface of TNS and plasma-TNS samples. According to Moulder et al. [32], the deconvolution of the O1s peak reveals three different oxygen atom states. The component located at the binding energy of 530.3 eV (O1) corresponds to oxygen O^2−^ in the TiO_2_ lattice structure; the component at 531.4 eV (O2) is often related to -OH groups; the component at 532.7 eV (O3) is typically associated with NO_x_ or H_2_O groups [34].

XPS spectra of the TNS surface showed that most of the oxygen was bonded in the form of an oxide (O1), and the proportion of -OH bonds was smaller (Figure 2G). This generally indicates that oxygen atoms are more likely to form TiO_2_ on the sample surface. In contrast to the TNS surface, a large number of O2 components were observed on the surface of plasma-TNS samples. These were related to the -OH group and caused by the interaction of energetic reactive oxygen species between the plasma and the material surface. As a result, the material surface hydrophilicity was significantly improved, consistent with the contact angle measurement results. The important O3 component typically associated with NO_x_ groups was detected on the surface of plasma-TNS samples; this was due to the Reactive Nitrogen Species (RNS) dominating the gas-phase chemistry during plasma treatment [35,36].

### 2.2. Biofilm Decontamination

**Figure 3:**
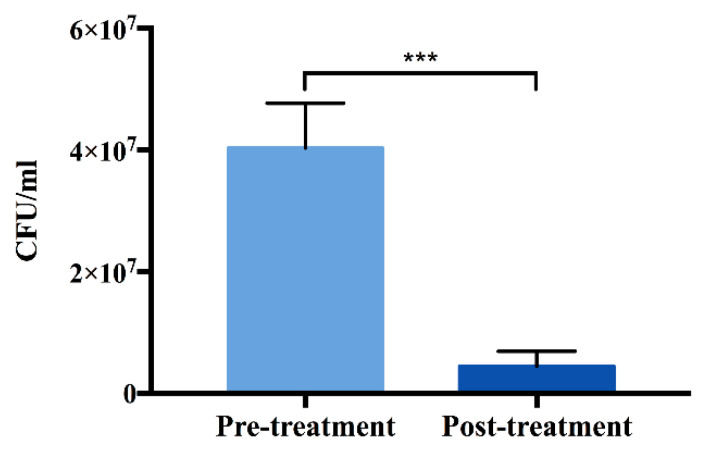
Effect of plasma treatment on decontamination. Data shown are the means ±SD (*n* = 3). *** *p* < 0.001.

In order to assess the efficiency of decontamination by plasma treatment, *Staphylococcus aureus (S. aureus)* culture was incubated on the surface of TNS for 24 h and allowed to develop into a biofilm. The surfaces were then exposed to plasma treatment. The results of inactivation demonstrate that plasma treatment could significantly decrease the *S. aureus* viability (Figure 3).

### 2.3. Determination of Intracellular Reactive Oxygen Species (ROS)

**Figure 4:**
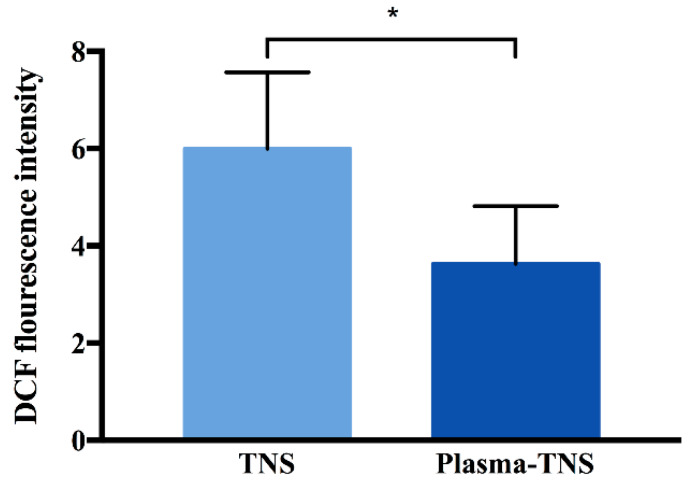
Determination of intracellular reactive oxygen species of rat bone marrow mesenchymal stem cells attached to the TNS and plasma-TNS disks. Data shown are the means ±SD (*n* = 3). * *p* < 0.05.

Furthermore, as shown in Figure 4, the level of intracellular ROS on the surface of TNS was significantly higher than that on the surface of plasma-TNS samples after incubation for 24 h, while there was no significant difference in the number of adhered cells between both types of surface.

### 2.4. Cell Adhesion and Morphology

**Figure 5:**
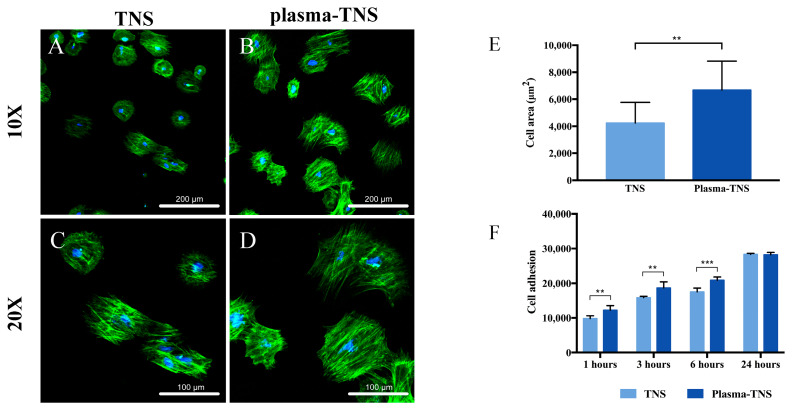
Morphological analysis of rBMMSCs attached to the (**A**,**C**) TNS and (**B**,**D**) plasma-TNS disks, (**E**) cell area, values were presented as mean ±SD of three representative images measured as per the surface of three samples in each group, (**F**) cell adhesion on TNS and plasma-TNS disks at 37 °C. Data shown are the means ±SD (*n* = 3). *** *p* < 0.001; ** *p* < 0.01.

After 24 h of incubation, cell morphology staining using phalloidin and DAPI showed that the cells adhered to plasma-treated surfaces had a higher cell area than those adhered to untreated surfaces (Figure 5A–E). The increase in initial cell adhesion and the changes in cell morphology likely contributed to the super-hydrophilicity of the surface produced by the plasma treatment [37] in accordance with the hydrophilicity analysis results. Moreover, the CellTiter-Blue^®^ Cell Viability Assay was used to evaluate the adhesion of rat bone marrow mesenchymal stem cells (rBMMSCs) to TNS and plasma-TNS samples. The results showed higher numbers of cells adhered to the surface of plasma-TNS samples at 1, 3, and 6 h (Figure 5F).

### 2.5. Osteogenic Activity of Rat Bone Marrow Mesenchymal Stem Cells (rBMMSCs)

**Figure 6:**
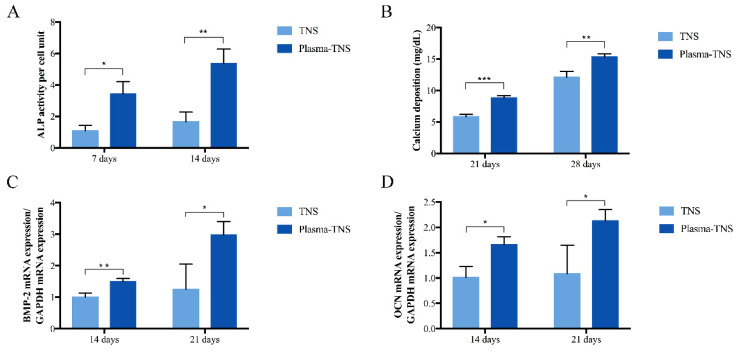
(**A**) Alkaline phosphatase activity, (**B**) calcium deposition, (**C**) bone morphogenetic protein 2 (BMP-2) and (**D**) osteocalcin (OCN) in cells grown on sample disks. Data shown are the means ±SD (*n* = 3). *** *p* < 0.001; ** *p* < 0.01; * *p* < 0.05.

ALP activity, which is a biochemical marker of osteoblast activity and cell phenotype in the early stage of cell differentiation and bone formation, was higher at seven and 14 days in the plasma-TNS group than in the TNS group (Figure 6A). Compared to the TNS group, the plasma-TNS group showed higher calcium deposition (a marker of extracellular matrix mineralization) at 21 and 28 days (Figure 6B). Gene expression of the bone morphogenetic protein (BMP) and osteocalcin (OCN), which are representative osteogenic differentiation products, were significantly higher in cells grown on plasma-TNS than in those cultured on TNS samples (Figure 6C,D). These results indicated that the differentiation activity of rBMMSCs was significantly promoted by surfaces treated with plasma.

### 2.6. Evaluation of the Bone Morphogenesis Around the Implant In Vivo

**Figure 7:**
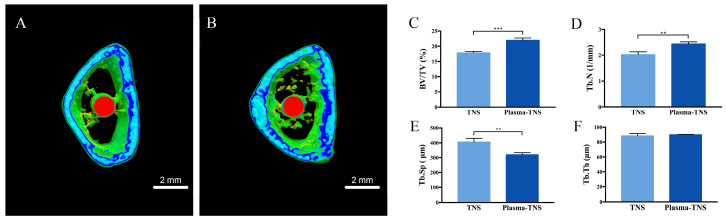
Reconstructed three-dimensional microcomputed tomography transverse slices of rat femurs containing TNS (**A**) and plasma-TNS (**B**) implants. The implant, cortical bone, and cancellous bone are shown in red, blue, and green, respectively. (**C**) Bone volume to total volume ratio (BV/TV), (**D**) mean trabecular number (Tb.N), (**E**) mean trabecular separation (Tb.Sp), and (F) mean trabecular thickness (Tb.Th) around implants after eight weeks. Data shown are the means ± SD (*n* = 3). *** *p* < 0.001; ** *p* < 0.01.

Bone formation activity around the TNS implant and the plasma-TNS implant was evaluated using a rat femur model. More trabecular microarchitecture was observed in the region of the plasma-TNS surface than in that of the TNS surface (Figure 7). Furthermore, the ratio of bone volume to total volume (BV/TV), mean trabecular number (Tb.N), and mean trabecular thickness (Tb.Th) were significantly higher in the plasma-TNS samples, indicating that the plasma-treated implants promoted osteogenesis activity (*p* < 0.01). Mean trabecular separation (Tb.Sp) was lower in the plasma-TNS implant than in the TNS implant (*p* < 0.01).

**Figure 8:**
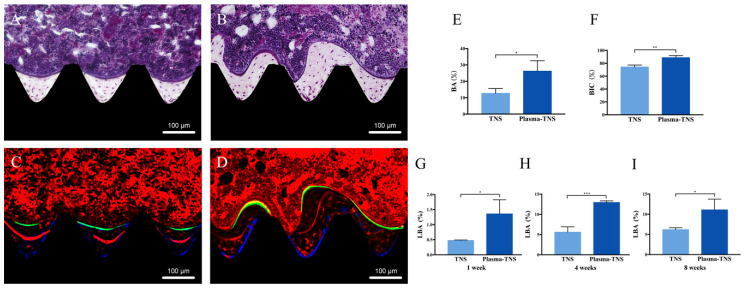
Villanueva staining of bone tissues around (**A**) TNS and (**B**) plasma-TNS implants. Fluorescence labeling of new bone and mineralization around (**C**) TNS and (**D**) plasma-TNS implants. (**E**) Bone area ratio (BA) and (**F**) bone-implant contact (BIC) of TNS and plasma-TNS implants. Fluorescently labeled bone area (LBA) after (**G**) one week, (**H**) four weeks, and (**I**) eight weeks. Data shown are the means ±SD (*n* = 3). *** *p* < 0.001; ** *p* < 0.01; * *p* < 0.05.

Furthermore, a longitudinal section was used to evaluate new bone formation around the implant. As shown in Figure 8, newly formed bone was observed around the plasma-TNS implant than the TNS implant. Quantitatively, the histomorphometric analysis showed that bone area ratio (BA) and bone-implant contact (BIC) were significantly higher around the plasma-TNS implants than around the TNS implants (Figure 8E,F). In addition, the newly formed bone around the implant was labeled with oxytetracycline hydrochloride (blue) at one week, alizarin red S (red) at four weeks, and calcein (green) at eight weeks. The labeled bone area between the implant interface and the labeled bone area at weeks 1, 4, and 8 was significantly higher in plasma-TNS implants than in TNS implants (Figure 8G–I).

## 3. Discussion

The surface morphology and chemical composition on the surface with nanostructures of titanium play a crucial role in mimic natural bone tissue and soft tissues to promote the bone healing process, which has been widely reported in the literature [15]. According to our previous experiments, the homogeneous nanoporous structures on the titanium surface generated by alkali treatment enhanced osseointegration, contributing to its excellent hydrophilicity and roughness compared with pure titanium [16,17]. Nevertheless, the resistance of nanostructured surfaces to biofilm is still insufficient, which could give rise to peri-implantitis, and more promptly, osseointegration, which is still a requirement in the early stage of implantation. In the present study, non-thermal atmospheric pressure plasma was employed to modify the nanostructured surface, and the effect of promotion of osteogenic activity and decontamination were evaluated comprehensively.

According to the nanotopographies observed by SEM and AFM, the sample surfaces revealed similar homogeneous nanoporous network structure whether treated by plasma or not, which indicated that plasma treatment does not destroy the geometry of the nanostructure on the sample surface and could thereby preserve the positive effect of the nanostructure on osseointegration. Additionally, XPS analysis confirmed that the chemical composition on the sample surface changed significantly after plasma treatment. Compared with the surface of TNS, a large number of polar oxygen groups (such as hydroxyl, carbonyl, and carboxyl groups) are formed after plasma treatment, which could enhance their hydrophilicity [35]. This is consistent with the analysis of the contact angle experiment, which determines that the hydrophilicity of the plasma-TNS surface has been further improved. Simultaneously, the increase of NOx after plasma treatment was also demonstrated by XPS analysis and may be due to the excessive gas-phase RNS produced by the plasma [38].

Moreover, the decrease of carbon and the formation of RONS were observed after plasma treatment, and were considered to be related to the effectiveness of the decontamination and the inhibition of early bacteria attachment and biofilm formation [39,40,41,42]. During plasma decontamination, bacteria are directly exposed to the plume of plasma that consists of abounding RONS, which induce membrane alterations and enzyme inhibition, prompt changes in membrane transport proteins, leading to the accumulation of more RONS, ultimately resulting in physiological dysfunction and cell death [43,44]. In addition, the results of the decontamination experiment showed that the plasma treatment applied to the biofilm-contaminated nanostructured surface could effectively eliminate the biofilm without destroying the nanostructure of the surface. Moreover, Lee et al. demonstrated that plasma treatment could decrease the carbon contamination to form a hydrophilic surface, thereby improving resistance to bacterial attachment and inhibiting the recolonization of biofilms, which would be highly beneficial from a clinical perspective [26].

According to the results of the cell adhesion experiment, plasma treatment did not induce cell apoptosis and even promoted cell attachment in the early stage of incubation [45]. Cell morphology analysis revealed higher cell areas on surfaces subjected to plasma treatment, which are likely to contribute to the increased hydrophilic character of plasma-treated samples [46]. Furthermore, the enhancement of osteogenic differentiation ability of cells was demonstrated by the results of ALP activity, calcium deposition, and bone morphogenetic protein 2 and osteocalcin gene expression. Simultaneously, animal experiments also revealed that plasma treatment contributed to the promotion of new bone generation and osseointegration comprehensively. According to the micro-CT, histological section, and fluorescent labeling analyses, plasma treatment had a significant positive effect on the promotion of osteogenesis. Notably, plasma treatment generated surfaces that strongly promoted new bone tissue formation at weeks 1 and 4 post-implantation, contributing to osteogenesis and confirming implant stability. These qualities play a decisive role in implantation success at the early stage of implantation, and our results thus support future research avenues utilizing and exploiting these promising qualities.

The results of XPS analysis revealed that plasma treatment significantly changed the chemical composition on the surface and led to an increase of polar oxygen groups (such as hydroxyl, carboxyl groups, etc.). Furthermore, we prefer to investigate the influences of these increased oxygen functional groups on the cells attached to the plasma-treated surface. Research on stem cell biology in recent decades has focused on that excessive intracellular ROS accumulation could damage proteins, lipids, DNA, and eventually lead to cell apoptosis [47,48], simultaneously, have clarified a variety of anti-oxidant and anti-stress mechanisms of stem cells [49,50]. Nevertheless, there is increasing evidence to support the opinion that intracellular ROS in redox homeostasis plays a critical part in maintaining stem cell self-renewal under some circumstances [51]. Indeed, stem cells are located in a state characterized by low levels of intracellular ROS, which are crucial to the regulation of the potential for self-renewal and stemness, while high levels of intracellular ROS effectively inhibit the ability of stem cells to self-renewal and differentiation [52,53,54]. Moreover, according to Ueno et al. reported, titanium surface pre-treated with UV light significantly reduced intracellular ROS and the expression of inflammatory cytokines, so that preventing the oxidative stress-induced DNA damage and promoting cell adhesion and spread [55]. Consequently, investigation of the link between intracellular ROS levels and altered chemical properties after plasma treatment may give insights into the mechanism by which plasma treatment promotes the osseointegration in the early stage of implantation. As the results have shown, cells attached to the plasma-treated surface exhibit a lower level of intracellular ROS compared with those on the untreated surface, while the increasing osteogenic activity. Recently, Gómez-Puerto et al. have demonstrated that oxygen functional groups could induce phosphorylation of forkhead box O3 (FOXO3) at serine 294, which was mediated by MAPK8 kinase, and its translocation to the nucleus. Simultaneously, activation of FOXO3 results in the downregulation of intracellular ROS through the activation of autophagy to maintain redox homeostasis during osteoblastic differentiation [56]. Furthermore, the overexpression of the FOXO3 gene in osteoblasts could decrease oxidative stress and osteoblast apoptosis, and increased bone formation rate [57]. We hypothesized that the oxygen functional groups on the plasma-treated surface might activate phosphorylation of FOXO3 to downregulate the oxidative stress state, which seems to be one of the possible mechanisms of the enhanced osteogenic activity after plasma treatment. Further experiments will be performed in the future to confirm whether the better osteogenic activity observed in the plasma-TNS group is due to the decrease of intracellular ROS induced by oxygen functional groups-mediated the phosphorylation of FOXO3.

In summary, it was demonstrated that plasma treatment could remarkably contribute to the enhancement of osteogenic activity and decontamination of the surface. Through the evaluation of the surface characteristics and biofilm decontamination, we confirmed that plasma treatment could eliminate the contamination without destroying the beneficial nanostructure of the surface. Following the results of osteogenic activity experiment both in vitro and in vivo, we also verified that the surface after plasma treatment is highly beneficial for the enhancement of osteoblast differentiation and early osteogenic activity. Additionally, owing to the excellent effect of simultaneous re-establishment of a highly hydrophilic surface suitable for osseointegration and elimination of the biofilm, and the clinically friendly advantages of plasma treatment, such as handheld, smooth operation, low cost, the wide-spread application of plasma is expected to be favorable either as a treatment immediately before implantation or as a therapeutic strategy for peri-implantitis. Furthermore, this study demonstrated that the roughness of nanostructured surface was not changed by plasma treatment, which could be of great significance for the combined application of this plasma treatment and other materials with the nanostructure. In future experiments, we will also establish an infection animal model and comprehensively evaluate the efficiency of plasma treatment on infected implants, as a novel therapeutic strategy for peri-implantitis.

## 4. Materials and Methods

### 4.1. Sample Preparation

Pure grade 2 titanium disks (15 mm in diameter and 1 mm in thickness) and titanium screw implants (1.2 mm in external diameter and 12 mm in length) were prepared by mechanical processing (Daido Steel, Osaka, Japan) to evaluate surface characteristics and for the animal study, respectively. The disks were then polished with incremental SiC abrasive papers (800#, 1000#, and 1500#). All samples were ultrasonically rinsed with acetone, ethanol, and deionized water (10 min each) and dried at room temperature overnight. All samples were immersed in a 10 M NaOH solution at 30 °C for 24 h, washed with ion-exchanged water (200 mL) several times until the conductivity of the solution reached 5 μS/cm^3^, and then dried at room temperature overnight to establish porous, homogeneous, and uniform nanonetwork structures (TNS) on the titanium surface.

### 4.2. TNS Plasma Treatment

Plasma treatment was performed using a non-thermal atmospheric pressure handheld plasma device (Piezobrush^®^ PZ2, Relyon Plazma GmbH, Regensburg, Germany) that utilized piezoelectric direct discharge technology. Half of the samples were treated at room temperature with plasma-induced by active gas at atmospheric pressure for 30 s. The distance between the jet exit and samples was set to 5 mm to ensure that the samples were wholly immersed in the plasma plume emerging from the nozzle. Plasma-treated TNS was tested in the experimental group, while the control group was untreated.

### 4.3. Surface Characterization

TNS and plasma-TNS surface topography were evaluated by SEM (S-4800, Shimadzu, Kyoto, Japan) with 10 kV accelerating voltage. AFM (SPM-9600, Shimadzu Co., Tokyo, Japan) was utilized to assay the mean average surface roughness (Ra), mean peak-to-valley height (Rz), surface profiles and three-dimensional surface topography of the samples. The sample chemical composition was determined by XPS (Kratos Axis Ultra, Shimadzu, Japan). Sample surface wettability was evaluated using a contact angle measurement system (VSA 2500 XE; AST Products, Billerica, MA, USA).

### 4.4. Biofilm Decontamination

*S. aureus* culture was prepared from a single colony inoculated into 5 mL of Tryptic soy broth (TSB) medium and incubated for 16 h at 37 °C. One milliliter of bacterial suspension, which was adjusted to a concentration of 1 × 10^5^ CFU/mL by adding fresh TSB medium, was added to the surface of TNS, resulting in biofilm formation after 24 h of incubation. Then, the bacterial suspension was removed, and samples were prepared for subsequent plasma treatment by rinsing with phosphate buffer saline (PBS) solution to remove nonadherent bacteria. Following the plasma treatment, samples were transferred into a sterile test tube containing 5 mL of TSB medium and vortexed for 2 min to dislodge the formed biofilm. The quantification of bacteria contained in the solution was performed by the plate-counting method [58].

### 4.5. Cell Culture

The rBMMSCs were obtained from the femurs of 8-week-old Sprague-Dawley rats (SHIMIZU Laboratory Supplies Co., Kyoto, Japan). Cells were cultured in growth medium containing minimal essential medium (Nacalai Tesque Inc., Tokyo, Japan), 10% fetal bovine serum (Nacalai Tesque Inc.), and antibiotic-antimycotic mixed stock solution (Nacalai Tesque Inc.) in a 5% CO_2_ humidified incubator at 37°C. The medium was changed every 3 days.

### 4.6. Cell Morphology

After 24 h of incubation, samples were washed with PBS, fixed by incubating with 4% paraformaldehyde solution for 20 min, permeabilized with 0.2% (*v*/*v*) Triton X-100 for 30 min, incubated with Blocking One reagent (Nacalai Tesque, Kyoto, Japan) for 30 min, and then stained with Alexa Fluor 488-phalloidin (Invitrogen/Life Technologies) and DAPI at 37 °C in the dark for 1 h. A confocal laser scanning microscope (LSM700; Carl Zeiss) was used to evaluate the F-actin and cell nuclei of adherent cells. A total of 30 cells randomly were selected from three representative images measured in per surface of three samples in each group, according to the recent report. And ImageJ software was used to the fluorescent image analysis.

### 4.7. Cell Adhesion

The rBMMSCs were seeded onto the specimens at an initial density of 4 × 10^4^ cells/cm^2^ and allowed to attach for 1, 3, 6, and 24 h. Following incubation at 37°C, the nonadherent cells were removed by washing with PBS (Nacalai Tesque, Inc.) and cultured with 300 μL of diluted CellTiter-Blue^®^ Reagent (50 μL CellTiter-Blue^®^ Reagent diluted in 250 μL PBS). After an additional 1 h of incubation, the fluorescence intensity was measured with a microplate reader (SpectraMax M5; Molecular Devices, Sunnyvale, CA, USA) according to the manufacturer’s protocol.

### 4.8. Determination of Intracellular ROS

Generation of intracellular ROS was analyzed by utilizing the oxidation-sensitive fluorescent probe 2’,7’-dichlorofluorescein diacetate (DCFH-DA, Sigma, St. Louis, MO, USA). Following incubation for 24 h, the cells washed with PBS, and incubated with 10 mM DCFH-DA for 30 min at 37 °C. Then, cells were washed twice with PBS, detached with 50 µL trypsin (0.25%), and diluted with 50 µL PBS. Fluorescence was then measured at excitation/emission wavelength of 485/528 nm using a fluorescence microplate reader, according to the manufacturer’s instruction.

### 4.9. Alkaline Phosphatase (ALP) Activity

In order to evaluate ALP activity, 4 × 10^4^ cells were seeded on specimens and cultured in α-MEM containing 10% fetal bovine serum, antibiotic-antifungal agent, 10 mM glycerophosphate (Wako Pure Chemical Industries, Osaka, Japan), and 10 nM dexamethasone (Nacalai Tesque). The differentiation medium was changed every 3 days. Following 7 or 14 days of incubation, samples were washed with PBS, and cells that had attached to the sample surface were dissolved with 300 μL of 0.2% Triton X-100. ALP activity was evaluated by an alkaline phosphatase luminometric enzyme-linked immunosorbent assay (ELISA) kit (Sigma-Aldrich) in accordance with the manufacturer’s instructions. A PicoGreen dsDNA analysis kit (Invitrogen/Life Technologies) was utilized to evaluate the DNA content. The amount of ALP was normalized to the amount of DNA in each cell lysate.

### 4.10. Extracellular Matrix Mineralization

Following 21 or 28 days of incubation, calcium deposition in the extracellular matrix was measured after dissolution with 10% formic acid. Calcium content was quantified and calculated using a Calcium E-test Kit (Wako Pure Chemical Industrials Ltd.) according to the manufacturer’s instructions.

### 4.11. Osteogenesis-Related Gene Expression

Expression of osteogenesis-related genes was assessed using a real-time TaqMan RT-PCR assay (Life Technologies, Carlsbad, CA, USA). Total RNA was extracted using a RNeasy Mini Kit (Qiagen, Venlo, the Netherlands), and 10-μL aliquots of each RNA sample were reverse transcribed into cDNA utilizing a Prime Script RT Reagent kit (TaKaRa Bio, Shiga, Japan). The mRNA levels of osteogenesis-related genes for bone morphogenetic protein 2 (*Bmp 2*) and bone gamma-carboxy glutamic acid-containing protein (*OCN*) were investigated using a Step One TM Plus RT-PCR System (Life Technologies). Relative gene expression levels in each group were normalized to that of the glyceraldehyde 3-phosphate dehydrogenase (*GAPDH*) housekeeping gene.

### 4.12. Animal Model and Surgical Procedures

The animal experiment was performed according to the ethical principles of the National Animal Care Guidelines and was approved by the Medical Ethics Committee of Osaka Dental University, Japan (approval no. 19-06002, 16 August 2019). Eight-week-old male Sprague-Dawley rats (Shimizu Laboratory Supplies Co., Kyoto, Japan) weighing 180–200 g were used in this study. The rats were randomly divided into two groups, with eight rats in each group. Surgical procedures used in this study were previously described [59]. After general anesthesia and surgical cleaning, a 10-mm longitudinal incision was made along the medial side of the knee joint of the right hind leg. The patella and extensor mechanism were then dislocated to expose the distal femur. A 1.2-mm hole was drilled into the intercondylar notch using a dental burr with sterilized saline irrigation. Screws were implanted into the prepared channels, the knee joint was restored, and the incision was sutured. Gentamicin (1 mg/kg) and buprenorphine (0.05 mg/kg) were injected for 3 days after surgery to prevent post-surgical infection and decrease postoperative pain.

### 4.13. Sequential Fluorescent Labeling and Microcomputed Tomography

Polychrome sequential labeling of bone via intraperitoneal injection of fluorescent dyes was employed to determine the process and characteristics of new bone formation and mineralization after implantation according to the following timetable: rats were injected with 25 mg/kg oxytetracycline hydrochloride (Sigma-Aldrich, USA) at 1 week after implantation, with 30 mg/kg alizarin red S (011-01192, Wako, JP) at 4 weeks, and with 20 mg/kg calcein (340-00433, Wako, Japan) at 8 weeks. Rats were then anesthetized and euthanized at 8 weeks, and the right femurs including the implants were placed in a saline solution immediately after dissection and scanned with an SMX-130CT microcomputed tomography (micro-CT) scanner (Shimadzu) operated at 90kV and 40 μA with a copper filter. Three-dimensional reconstruction models were obtained using morphometric software (TRI/3D-BON; Ratoc System Engineering, Tokyo, Japan). The region of interest was defined as 2 mm below the highest point of the growth plate and extending 500 μm around each implant. The bone volume fraction (BV/TV), mean trabecular number (Tb.N), mean trabecular thickness (Tb.Th), and mean trabecular separation (Tb.Sp) were quantified to assess bone regeneration.

### 4.14. Histology of Sequentially Labeled Sections

After the micro-CT scan, implanted femurs collected at 8 weeks were stained utilizing the Villanueva method to evaluate bone generation. All histomorphometric and fluorescence characteristics of the sections were analyzed using a BZ-9000 digital cold illumination microscope (Keyence Co., Osaka, Japan) and a laser scanning microscope (Carl Zeiss, Oberkochen, Germany), respectively.

The excitation and emission wavelengths were 351/460 nm for oxytetracycline hydrochloride (blue), 543/617 nm for alizarin red S (red), and 488 nm/517 nm for calcein (green), respectively. Bone area, BIC, and labeled bone area were assessed utilizing ImageJ software in a 200× field around the implant.

### 4.15. Statistical Analysis

All data were expressed as the mean ± standard deviation. Each experiment was repeated three times, and all results were compared in SPSS 26.0 by Student’s t-test; P< 0.05 was considered statistically significant.

## 5. Conclusions

Plasma treatment of titanium surfaces with nanonetwork effectively changed the chemical composition of the sample surface, which further improved implant hydrophilicity and facilitated cell attachment and extension; significantly accelerated new bone generation and improved osseointegration in the early stage of implantation. Furthermore, the plasma treatment efficiently decontaminated the implant surface while preserving the nanoscale morphology of the TNS surface and generated a surface beneficial to osteogenesis at the same time, which could be used as a novel approach for immediate treatment before implantation or as the therapeutic method of peri-implantitis. Moreover, the elucidation of the effect on cell adhesion, differentiation, and decontamination by plasma treatment has imparted meaningful advice for future research and the development of plasma-based therapeutic strategies.

## Figures and Tables

**Table 1:** Roughness values of the TNS and plasma-TNS. Data shown are the means ±SD (*n* = 3).

Group	Parameters	
	Ra (nm)	Rz (nm)
TNS	24.71 ± 7.14	218.93 ± 89.48
Plasma-TNS	27.16 ± 5.01	233.90 ± 19.79

## Abbreviations

TNS	Titanate layer with nanonetwork structures
RONS	Reactive oxygen and nitrogen species
SEM	Scanning electron microscopy
AFM	Atomic force microscopy
XPS	X-ray photoelectron spectroscopy
rBMMSCs	Rat bone marrow mesenchymal stem cells
PBS	Phosphate buffered saline
ALP	Alkaline phosphatase activity
ELISA	Enzyme-linked immunosorbent assay
Micro-CT	Microcomputed tomography
FOXO3	Forkhead box O3
BV/TV	Bone volume to total volume ratio
Tb.N	Mean trabecular number
Tb.Sp	Mean trabecular separation
Tb.Th	Mean trabecular thickness
BA	Bone area ratio
BIC	Bone–implant contact
BMP	Bone morphogenetic protein
OCN	Osteocalcin
